# Postoperative analgesic efficacy of ear acupuncture in patients undergoing abdominal hysterectomy: a randomized controlled trial

**DOI:** 10.1186/s12871-020-01187-4

**Published:** 2020-11-09

**Authors:** Hamdy A. Hendawy, Mohamed E. Abuelnaga

**Affiliations:** 1 Faculty of Medicine, Suez Canal University, Ismailia, Egypt; 2 Department of Anesthesia, Intensive Care and Pain Management, Faculty of Medicine, Suez Canal University, P.O. Box: 41522, Ard Elgameiat, Ismailia City, Egypt

**Keywords:** Electric ear acupuncture, Post-operative pain relief, Abdominal hysterectomy

## Abstract

**Background:**

Numerous studies have revealed that acupuncture can increase the somatic pain threshold. Electro-acupuncture (EA) can help pain-relieving with minimal physiologic disturbance. Various painful disorders, as well as pain following various surgeries, like cesarean section, gastrostomy, and enterectomy were managed properly with acupuncture. Therefore we studied the postoperative analgesic effect of EA in patients undergoing abdominal hysterectomy.

**Methods:**

A randomized, prospective clinical trial study was carried out on 56 women undergoing hysterectomy under spinal anesthesia. Patients were allocated randomly to receive either spinal anesthesia and electric ear acupuncture (EEA group) or spinal anesthesia alone (control group). EEA was done by fine needles to anatomically defined 4 points of the ear: Shen Men Point, thalamus Point 26, Analgesia Point 3, and Uterus Point 58, and connected to EA therapeutic apparatus. After finishing surgery, the fine needles were substituted by permanent press needles to be removed after 24 hours. The primary outcome was the postoperative 24 h morphine consumption by patient-controlled analgesia, while secondary outcomes included Post-operative pain scores and postoperative 1st request of analgesia.

**Results:**

Total morphine consumption in the first 24 postoperative hours was obviously reduced in the EEA group versus the control group (mean ± SD:6.214± 2.1319 mg vs 15.714 ± 3.3428 mg, d = − 3.3886, 95% Confidence interval = − 4.2061,-2.5712, *p*-value =0.000). The postoperative pain scores were significantly reduced in the EEA group in comparison to the control group, with delayed 1st request of postoperative analgesia.

**Conclusions:**

Electric ear acupuncture provides postoperative analgesia, reducing morphine requirement and consequently its side effects.

**Trial registration:**

The trial was registered before enrolment of the first patient at the Pan African Clinical Trial Registry (www.pactr.org) database (PACTR201903770607799, Date of registration: 5th March 2019).

## Background

Acupuncture is considered as a traditional therapeutic technique with a long history of 4000 years. Electro-acupuncture (EA) is a new type of hand acupuncture that can deliver effective analgesia with a minute physiological disturbance [1, 2]. It was used effectively for pain management in different disorders including post-surgical pain following cesarean section, gastrostomy, and enterectomy [1, 3, 4]. Acupuncture and Transcutaneous electrical nerve stimulation (TENS) were showed to have valuable efficacy in increasing the somatic pain threshold [5]. Kitade and Hyodo [6] investigated the effect of auricular acupuncture stimulation on the threshold of somatic pain. They found the Shen-Men, lungs, sympathicus, and kidney points showed a significant pain threshold rise when compared to non-acupuncture auricular points. Even though the results differ greatly depending on the individual and body region, to some degree they elucidated that auricular acupuncture points had parallels in specific body regions and the authors concluded that auricular acupuncture stimulation can achieve an analgesic effect. It is believed that the human body encompasses energy pathways or channels representing the internal organs. Energy is thought to stream continuously through these pathways ending to the body surface with acupuncture points that work as gateways to these channels. Stimulation of these acupoints is claimed to activate the body’s inherent healing power [7].

Generally, acupuncture can boost physical and emotional health through the release of certain neurochemicals from the nervous system, as well as affecting cerebral areas responsible for reducing pain and anxiety [7, 8]. Acupuncture can help in postoperative pain reduction by altering the brain’s chemistry, increasing endorphins and neuropeptide Y levels, and reducing serotonin levels [7]. Systematic reviews revealed the effectiveness of acupuncture as a useful adjuvant for post-surgical pain relief. Randomized controlled trials have found that acupuncture can ameliorate postoperative pain, reduce the use of patient-controlled analgesia (PCA), and postoperative nausea and vomiting [8]. Several publications have found the analgesic effectiveness of body acupuncture in gynaecologic surgery including hysterectomy [9–12]. Tsang et al. reported that a single session of auricular TENS delivered 24 hours after surgery was effective in reducing post-hysterectomy pain for at least 30 min [13]. This study aims to investigate the role of preoperative electric ear acupuncture (EEA) in reducing post-operative pain in patients undergoing hysterectomy by fine acupuncture needles (10 mm × 30 gauge) for four points of ear acupuncture. We hypothesize that EAA will reduce the required postoperative analgesia following abdominal hysterectomy.

## Methods

The current study is a single-blinded randomized controlled clinical trial. After Obtaining approval by the Hospital Ethics Committee, and Written informed patient consent with an explanation regarding the purpose, methods, effects, and complications of techniques, 56 patients were randomly allocated into two equal groups using simple random tables generated from a computer software program (http://www.randomizer.org). The allocation sequence was concealed in opaque numbered envelopes. The outcome assessor was another anesthesiologist who was blind to the study group. Inclusion criteria included patients aged between 21-60 years old and have an American society of anaesthesiologist (ASA) physical status I & II. Patients who have contraindications for spinal anesthesia as (infection at the site of puncture, coagulopathy, platelet abnormalities, and concurrent treatment with anticoagulant drugs), allergy to morphine, and/or bupivacaine, morbid obesity (body mass index (BMI) > 35 Kg/m^2^), psychological disease, narcotic abuse, prolonged use of sedatives or opioid drug use, contraindications for ear acupuncture (infection at the acupuncture site) and patients refused to participate in the study were excluded. The primary objective was to assess the influence of auricular acupuncture regarding the postoperative analgesic requirement in patients scheduled for abdominal hysterectomy, while secondary objectives were intraoperative hemodynamic parameters, Post-operative pain scores, and Postoperative 1st request of analgesia.

### The 56 patient were randomized into two equal groups:-

- **Group I (EEA group):** 28 females received electric ear acupuncture after spinal anesthesia.
- **Group II (control group):** 28 females received spinal anesthesia without ear acupuncture.

#### Preoperative visit to the chosen patients was done where

Explanation of the whole procedure including the expected benefits and complications was done. All Patients were instructed preoperatively for the use of the PCA apparatus.

### A) Pre-operative assessment

The patient’s age, ASA status, and BMI were recorded. Medical history was reviewed to rule out excluded patients from the study. General examination, vital signs: heart rate (HR), blood pressure (BP), temperature, chest, heart, abdominal examination, and airway evaluation were done. Investigations done were complete blood count, random blood sugar, bleeding profile as prothrombin time, International Randomized Ratio, and partial thromboplastin time.

### B) Intra-operative measures

The patient was reassured and the procedure was explained to her. Airway devices and anesthesia machines, ventilators, flowmeters, and equipment were checked promptly. Routine Monitoring equipment (Datex-Ohmeda®) was attached to the patient including 3 leads electrocardiogram (ECG), non-invasive BP, and pulse oximetry. An 18- gauge intravenous cannula was inserted into the patient’s forearm and 500 mills of intravenous (I.V.) fluid (ringer acetate) were administrated. No premedications were given. All patients in both groups were given spinal anesthesia by 25 G needle with hyperbaric bupivacaine 0.5% (15-20 mg) according to the patient’s height, in the L4- L5 intervertebral space. During spinal injection, no supplementary opioids were given; the level of anesthesia was assessed by pin-prick every 5 min in the first 20 min till it reached T4-T6 which would be considered sufficient to start the surgery.

### The procedure of acupuncture

Acupuncture was done based on our institutional experience. EEA was done by fine needles (10 mm x 30guage) to the anatomically defined 4 points of ear acupuncture which are called; Shen Men Point (Divine Gate Point) or point 55 which is a sedative analgesic anti-inflammatory point, thalamus Point 26 which is an analgesic point in acute and chronic severe pain, Analgesia Point 3, and Uterus Point 58 [7, 8].

**Fig. 1:**
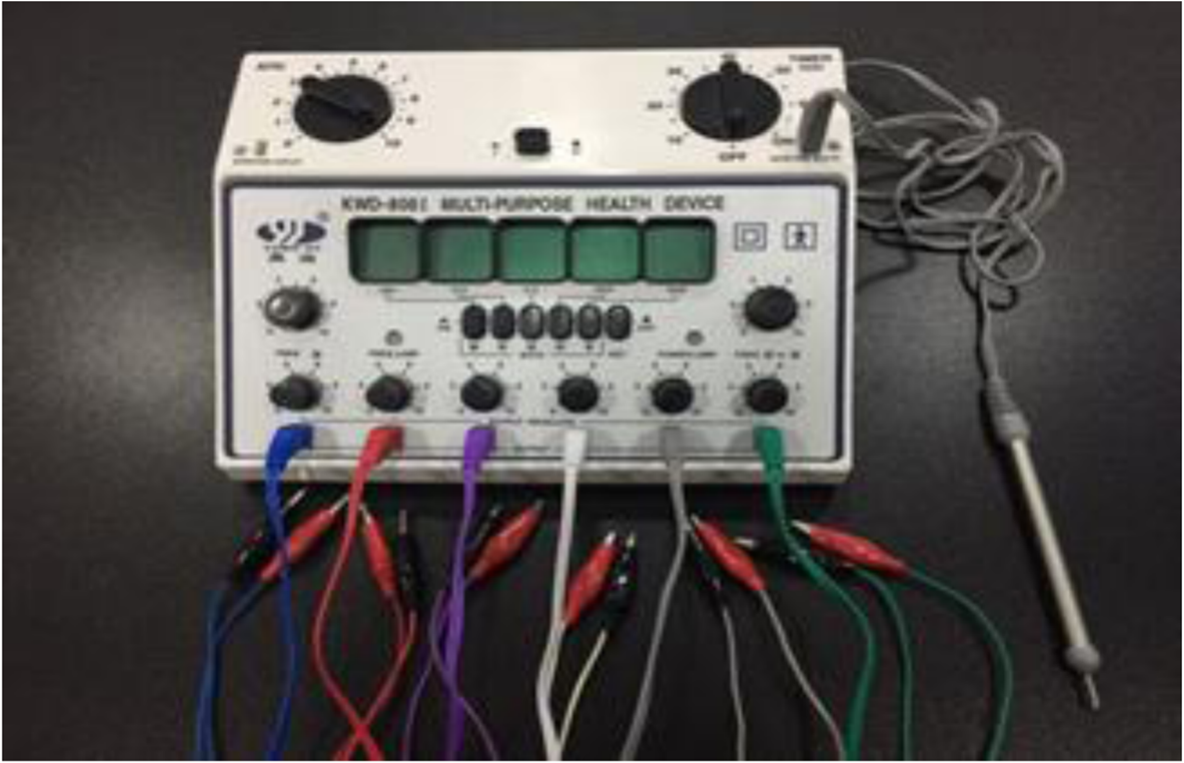
KWD-808I MULTIPURPOSE HEALTH DEVICE is connected to grey acupoint detecting pen for acupoint detection APD and six electrode cords with 6 different colors for electro-acupuncture

Then four pre-sterilized single-use acupuncture needles were inserted in the predetermined acupoints and connected to clamps of electrode cords that were connected to the electro-acupuncture therapeutic apparatus (KWD-808I MULTIPURPOSE HEALTH DEVICE, Changzhou Yingdi Electronic Medical Device Co., China. (Fig. 1).

The EA device was switched on and adjusted according to the instruction manual for use to provide continuous-wave with a frequency of 2 Hz and output impulse amplitude of 6-10 V according to the patient’s ability. At the end of the surgery, the electro-acupuncture device was switched off and the fine needles were replaced by press needles in the same acupoints to be removed after 24 hours.

#### Measurements and postoperative care

All intraoperative and postoperative recordings were done by another anesthesiologist who was blind to the allocation group. Vital signs (BP, HR, respiratory rate) and oxygen saturation were recorded and analyzed every 5 min in the first 20 min, then every 20 min for the rest of the surgery. At the end of the operation, the duration of surgery and spinal level were recorded for both groups, and Diclofenac potassium 75 mg was administered to all patients by intramuscular injection. Postoperative BP and HR were recorded immediately after transfer to the post-anesthesia care unit (PACU), then after 1, 3,6,12, and 24 hours. Post-operative pain assessment was done by visual analog scale .when the reported visual analog scale (VAS) was 3 or more, a loading dose of morphine 0.05 mg/kg was administered via slow I.V. route. Then, a PCA was administered. The PCA pump was loaded with 1 mg / ml of morphine and set to deliver on-demand doses of 2 ml with 10 min lockout intervals and 4 h limit of 20 mg. No background infusion was allowed. The pain score was reassessed at the end of the 1st postoperative 24 hours. The time to the 1st postoperative request of analgesia was recorded using the PCA delivery system. The total amount of morphine consumption over the 1st 24 hours postoperatively was recorded. Side effects associated with morphine consumption as nausea, vomiting, and over sedation were recorded. Nausea and vomiting were assessed using a 5 point scale from zero to four, where 0 is no nausea or vomiting, 1 = mild nausea, 2 = severe nausea, 3 = vomiting once, 4 = vomiting more than one. These assessments were performed in the PACU immediately after discharge and after 1, 3, 6, 12, and 24 h postoperatively. Side effects associated with auricular acupuncture-like bleeding, excessive pain, fainting, broken needles, and local infection were recorded.

### Statistical analysis

The statistical analysis was performed using IBM SPSS Statistics® 22 for Windows 10 operating system**.** Normality was assessed using the Kolmogorov–Smirnov test and visual inspection of histograms. For demographic and baseline comparison, the standardized difference was calculated using a web-based effect-size calculator [14]. 2-tailed t-test was used to analyze the continuous variables (hemodynamic parameters, pain score, 1st request of analgesia, 24 hours analgesic consumption) between the two studied groups while chi-square test was used for categorical and dichotomous variables (incidence of nausea, vomiting and level of the block at end of surgery). Values were expressed as mean ± standard deviation (SD) for continuous variables or as a percentage of the group from which they were derived (categorical variables). *P*-value < 0.05 was considered significant. Presentation of the statistical outcomes in the form of tables and graphs was performed using the “Microsoft Office Excel® 2010” program.

24 patients per group were needed to detect 5.39 mg difference between the means of total 24 hours postoperative morphine consumption in patients undergoing cesarean section between electro-acupuncture and control groups, at a standard deviation of 5.18 [15], with 95% power and a level of significance 5%. After considering a 20% dropout rate, the required sample size was 56 patients (28 patients/group).

## Results

**Fig. 2:**
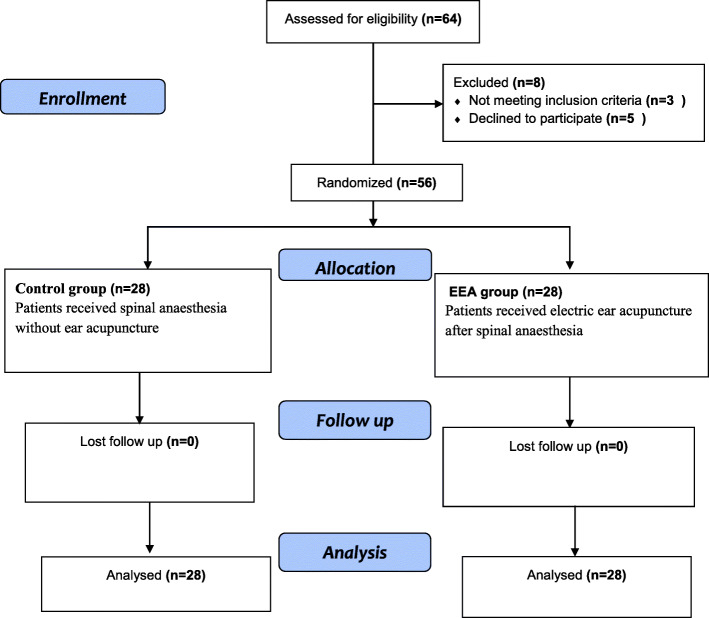
Flow chart of patient’s participation progress throughout the study

Fifty-six patients were randomly assigned for two equal groups EEA group and the control group (Fig. 2)

The two groups were comparable in age, BMI, ASA status, history of chronic illness, duration of surgery, and level of the block at end of surgery. The duration of surgery was about 80 min in both groups. (Table 1) no statistically significant difference was found between the 2 groups as regard intraoperative hemodynamics.

**Table 1:** Demographic characteristics among the studied patients

Demographic characteristics			EEA group (*n* = 28)	control group (*n* = 28)	*d*	95% C.I.	p
Age	Mean ± SD		49.857 ± 3.9320	49.643 ± 3.8318	0.0551	− 0.4688,0.579	0.837 (NS)
BMI	Mean ± SD		27.02 ± 3.401	28.38± 2.794	− 0.437	− 0.967,0.0931	0.108(NS)
ASA I ASA II	N (%)		16 (57.1%) 12 (42.9%)	16 (57.1%) 12 (42.9%)	0	−0.5292,0.5292	1.0(NS)
Chronic illness	Hypertension	N (%)	5 (17.9%)	4 (14.3%)	−0.0981	−0.6222,0.4261	0.716(NS)
	Diabetes mellitus	N (%)	7 (25.0%)	8 (28.6%)	0.0813	−0.4427,0.6054	0.763(NS)
Duration of surgery	Mean ± SD		79.96 ± 8.113	80.61 ± 6.190	−0.0901	−0.6142,0.434	0.74(NS)
Level of the block at end of surgery	T4 T6	N (%)	15 (53.6%) 13 (46.4%)	14 (50%) 14 (50%)	−0.0721	−0.5961,0.4519	0.789 (NS)

*NS* Non-significant difference (*P* > 0.05)

*p p*-value for comparing between the two groups

*d* standardized mean-difference effect size

*C.I* confidence interval

The time to the 1st request of analgesia was much delayed in the EEA group versus the control group (mean ± SD: 268.893 ± 31.6044 vs 52.036 ± 9.5316 min) in both groups respectively with statistically significant difference (d = 9.2905, 95% CI = 7.4919, 11.089, *p*-value =0.000). Total post-operative 24 hours morphine consumption was markedly reduced in favor of the EEA group (6.214± 2.1319 mg) versus (15.714 ± 3.3428 mg) in the control group with statistically significant difference (d = − 3.3886, 95% CI = -4.2061,-2.5712,*p*-value =0.000). postoperative VAS score after 24 hours was lower in the favor of the EEA group in comparison to the control group (mean ± SD: 2.536 ± 1.1380VS 5.036 ± 1.3467) in both groups respectively with statistically significant difference (d = − 2.0053, 95%CI = -2.6474, − 1.3631, *p*-value =0.000). (Table 2).

**Table 2:** Postoperative time for the first analgesic request (mins), VAS at 24 hours postoperatively, and total analgesic consumption in the 1st 24 hours (mgs)

	EEA group (*n* = 28)	Control group (*n* = 28)	*d*	95% C.I.	*p*
First analgesic request (Mean ± SD)	268.893 ± 31.6044	52.036 ± 9.5316	9.2905	7.4919,11.089	0.000*
24 hours morphine consumption (mean ± SD)	6.214± 2.1319	15.714 ± 3.3428	−3.3886	−4.2061,-2.5712	0.000*
VAS at 24 hours (Mean ± SD)	2.536 ± 1.1380	5.036 ± 1.3467	-2.0053	-2.6474, −1.3631	0.000*

*statistically significant at *p* ≤ 0.05

*p p*-value for comparing between the two groups

*d* standardized mean-difference effect size

*C.I* confidence interval

There was a statistically significant difference(*p*-value < 0.05) between the two groups regarding the incidence of nausea at 1 h, 3 h, 6 h, and 12 h in the favor of the EEA group. (Table 3).

**Table 3:** Post-operative incidence of nausea and vomiting

		post-operative nausea			post-operative vomiting		
		EEA group (*n* = 28)	Control group (*n* = 28)	*p*	EEA group (*n* = 28)	Control group (*n* = 28)	*p*
PACU	N (%)	0	0	.	0	0	.
After 1 h	N (%)	0	7 (25%)	0.005*	0	0	.
After 3 h	N (%)	0	6 (21.4%)	0.01*	0	0	.
After 6 h	N (%)	0	8 (28.6%)	0.002*	0	4 (14.3%)	0.038*
After 12 h	N (%)	0	20 (71.4%)	0.000*	0	0	.
After 24 hr	N (%)	0	0	.	0	0	.

*statistically significant at *p* ≤ 0.05

*p p*-value for comparing between the two groups

Vomiting occurred in 4 patients of the control group at 6 h time point while it was not noted to occur in the EAA group with a significant difference between both groups (*p*-value =0.038). (Table 3). Over-sedation was not noted in both groups of the study. No complications were recorded from auricular acupuncture.

**Fig. 3:**
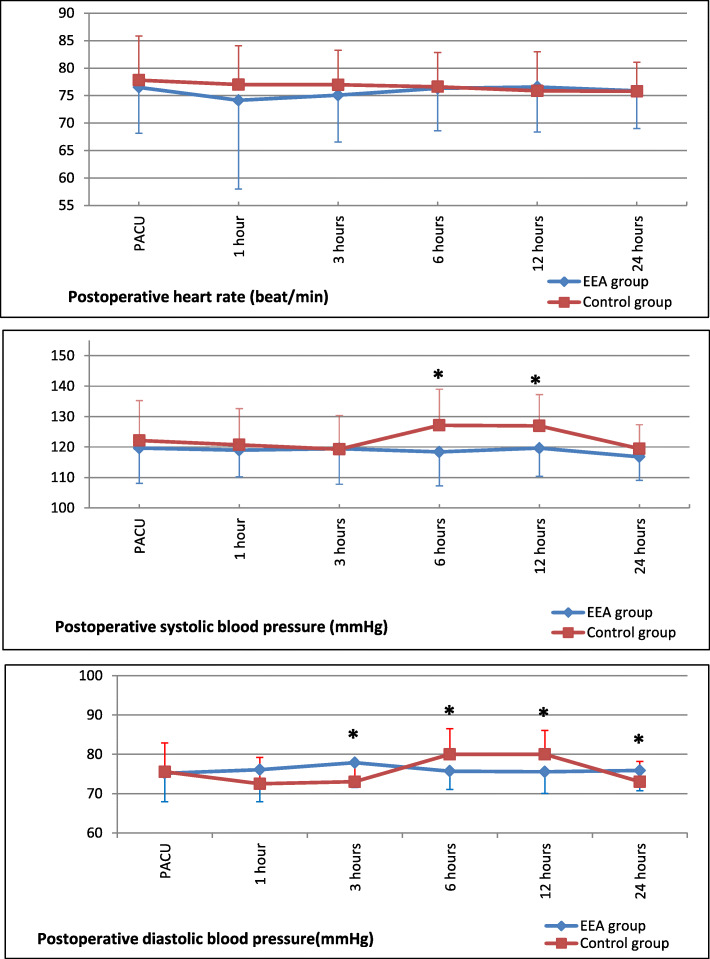
Postoperative hemodynamics in the study groups

Statistically, a significant difference was noted regarding postoperative systolic blood pressure changes between the two studied groups after 6 h and 12 h of surgery in favor of the EEA group. There was also a significant difference regarding the post-operative diastolic BP changes between the two groups after 3 h, 6 h, 12 h, and 24 hours after surgery in the favor of the EEA group. No statistically significant difference was noted as regards the postoperative HR between both groups at all time points. (Fig. 3).

## Discussion

Acupuncture and TENS were proven to increase the threshold of somatic pain. Ishimaru and colleagues examined how low flow electric acupuncture (LFEA) can influence pain threshold in the abdominal area; they advocated that LFEA can be helpful for analgesia following abdominal surgery [16].

The effect of LFEA and TENS on the deep pain threshold at the skin surface and deeper layers has been examined by a few studies [17, 18]. Kitade and colleagues found an increased pain threshold for 50 min following LFEA, with no identified increase in the levels of plasma β-endorphin or adrenocorticotropic hormone in the cerebrospinal fluid [19]. These results eliminate the role of β-endorphin in the analgesic effect of acupuncture. Because acupuncture analgesia was offset by naloxone, there is a probability that some types of endogenous opioid peptides are responsible for the analgesic effect of acupuncture. Ishimaru et al. showed that 30 min of LFEA produced analgesia [20]. They found increased levels of plasma β-endorphin [20, 21], with no change in plasma adrenocorticotropic hormone level [21]. This result matches what has been found in patients with post-operative pain [21].

Several mechanisms for acupuncture pain have been reported as the release of spinal natural opioids like dynorphin, endorphin, and encephalin, which are potent pain suppressors, increase in local blood flow that encourages the process of healing, gate control theory of pain, the endogenous release of adrenocorticotrophic hormones, myofibrillary entanglement relaxation, and creating a balance in the mesolimbic neural pain pathway [22].

In 2016, De Freitas et al., have reported in an animal study that acupuncture has a pre-emptive analgesic effect [23]. Sim et al. 2002 compared preoperative versus postoperative body EA for lower abdominal gynaecologic surgery. Preoperative acupuncture was found to be more effective in reducing postoperative morphine consumption in these patients [11].

In the current study, we decided to use preoperative EEA along with continuous postoperative acupoint stimulation by press needles to achieve the maximum analgesic effect. Our study demonstrated that the total postoperative PCA morphine consumption in the first 24 hours was reduced in the EEA group versus the control group; also the time of the first request for supplemental analgesia was delayed in the EEA group when compared with the control group.

Similar to our findings: In a prospective, randomized study done by WU Hung-Chien et al. [15] and conducted for 60 primigravida women scheduled for cesarean section under spinal anesthesia. Patients were allocated to one of three groups: control group, acupuncture group, and electro-acupuncture group. Acupuncture was started postoperatively after recovery from anesthesia and was applied for San yin Jiao (Sp6) body acupoint for 30 min. The results showed delayed first analgesic request in both the electro-acupuncture group (39.5 ± 16.9 min) and the acupuncture group (40.3 ± 13.8 min) versus the control group (29.0 ± 15.0 min). The total PCA morphine consumption during the first 24 postoperative hours was much reduced in the acupuncture group (10.66 ± 4.68) mg, and the electroacupuncture was (9.89 ± 5.18) mg than in the control group was (15.28 ± 4.99) mg [15]..

In our study, we had a delayed first morphine request and less total morphine dose which may be due to our usage of diclofenac potassium by intramuscular injection, the pre-emptive effect of acupuncture, and also acupuncture needles were replaced in the EEA group by press needles.

In our study, the VAS pain scores were lower in the EEA group versus the control group. Similarly, WU Hung-Chien, et al. [15] found that VAS pain scores were significantly lower in the auricular acupuncture group versus the control group at 0.5, 1, 1.5, and 2 hours in the postoperative period.

Similarly, Iacobone,m et al. 2014 [24], found less postoperative pain scores and reduced postoperative acetaminophen consumption in acupuncture treated groups versus the control group in patients undergoing thyroidectomy under general anesthesia.

Chen, C. C et al., 2015 [25], have investigated the effect of acupuncture on Pain Relief following total knee arthroplasty in a randomized controlled trial. The total amount of postoperative fentanyl consumption was found much reduced in the acupuncture group in comparison with the control group [mean (SD), 620.7 (258. 2) vs. 868.6 (319.3) μg, respectively, as well as delayed first request for fentanyl [median time, 89 vs. 37 min], in the acupuncture group and the control group respectively. Postoperative pain scores were much reduced in the acupuncture group versus the control groups at 2, 4, 8, 12, and 24 hours. Dingmann, J et al. 2017 [26], have found in their study the efficacy of acupuncture in reducing postoperative swallowing pain scores following tonsillectomy in patients aged 16 years or more.

In our study, there was a statistical difference between both groups as regards the incidence of nausea at some time points in the favor of the EEA group; also we found a significant difference between both study groups concerning the incidence of vomiting after 6 h in the favor of acupuncture group. This may be explained by a reduced total amount of 24 hours of morphine consumption in the acupuncture group in comparison to the control group.

Usichenk et al. 2004 [27] found that auricular acupuncture (AA) is effective in the treatment of various pain conditions. They conducted a blinded study to assess the analgesic effect of AA for pain management following total hip arthroplasty. The patients were randomly assigned to receive either true AA or sham acupuncture, with press acupuncture needles kept in place for 3 postoperative days. Thirty-six hours of analgesic requirement following surgery was lower in the AA group versus the control group. No difference was found between both groups as regard VAS Pain scores or analgesia-related adverse effects.

Chang et al. 2012 [28] have conducted a randomized controlled study to investigate the potential benefits of auricular acupressure for postoperative pain relief and ameliorating the passive range of motion in patients who underwent total knee replacement (TKR). Sixty-two patients were randomly allocated to either the acupressure group or the control group. The acupressure was performed three times a day for 3 days. The patients in both groups were found to have a comparable high level of postoperative pain scores. However, analgesic drug consumption was markedly reduced in the acupressure group versus the control group (*P <* 0*.*05), with markedly improved passive knee motion by the third postoperative day in patients treated with acupressure (*P* < 0.05) [28].

Lastly, we have some limitations. The analgesic effect found in the current study can be the result of either preoperative EEA, continuous post-operative acupoint stimulation by press needles, or combined effect of both techniques. Comparison between EEA and pressure acupuncture in the same acupoints is recommended in future studies. The other limitation in the current study was the use of a single postoperative non-opioid analgesic. In future studies, at least two non-opioid analgesics should be used to determine the real influence of acupuncture on postoperative analgesia requirements.

## Conclusion

Auricular acupuncture is a useful analgesic adjuvant as it reduced postoperative analgesic requirements following abdominal hysterectomy. It provided more patient satisfaction regarding postoperative pain control without any hazard.

## Data Availability

The datasets generated during and/or analyzed during the current study are available from the corresponding author on reasonable request.

## Abbreviations

EA: Electroacupuncture
EEA: Electric ear acupuncture
PCA: Patient controlled analgesia
TENS: Transcutaneous electrical nerve stimulation
ASA: American society of anesthesiologist
BMI: Body mass index
HR: Heart rate
BP: Blood pressure
PACU: Postanesthesia care unit
VAS: Visual analog scale
